# A Screening Mechanism Differentiating True from False Pain during Empathy

**DOI:** 10.1038/s41598-017-11963-x

**Published:** 2017-09-13

**Authors:** Ya-Bin Sun, Xiao-Xiao Lin, Wen Ye, Ning Wang, Jin-Yan Wang, Fei Luo

**Affiliations:** 1 CAS Key Laboratory of Mental Health, Institute of Psychology, Chinese Academy of Sciences, Beijing, 100101 P.R. China; 2 Department of Psychology, University of Chinese Academy of Sciences, Beijing, 100049 P.R. China

## Abstract

Empathizing with another’s suffering is important in social interactions. Empathic behavior is selectively elicited from genuine, meaningful pain but not from fake, meaningless scenarios. However, the brain’s screening mechanism of false information from meaningful events and the time course for the screening process remains unclear. Using EEG combined with principle components analysis (PCA) techniques, here we compared temporal neurodynamics between the observation of pain and no-pain pictures as well as between true (painful expressions and needle-penetrated arms) and false (needle-penetrated faces with neutral expressions) pain pictures. The results revealed that pain vs. no-pain information is differentiated in the very early ERP components, i.e., the N1/P1 for the face and arm pictures categories and the VPP/N170 for the facial expression category while the mid-latency ERP components, N2 and P3, played key roles in differentiating true from false situations. The complex of N2 and P3 components may serve as a screening mechanism through which observers allocate their attentions to more important or relevant events and screen out false environmental information. This is the first study to describe and provide a time course of the screening process during pain empathy. These findings shed new light on the understanding of empathic processing.

## Introduction

The sight of an elderly woman lying in the road with bruises and a grimace on her face elicits pity which urges you to rush to her and help her up. This scene is an example of the psychological phenomenon known as empathy, which is generally defined as the capacity to perceive and understand the emotions, feelings and inner states of another individual^1–3^. As a basic building block of human social interaction, empathy is believed to contribute greatly to cooperation, socialization and the development of morality^2, 4, 5^. Most everyone is affected when seeing others in pain, and as such, painful scenes are the most common and robust trigger for empathy^6–8^. Prior studies have shown that exposure to another’s pain can activate brain regions involved in the direct experience of pain^9–11^. Through this automatic matching mechanism, one can quickly react to another’s suffering.

It is well accepted that empathy is followed by prosocial behavior^12, 13^. In cases where subjective reports are unavailable, prosocial behaviors are treated as an index of empathy^14–16^. However, one may not react to all pain cues in the same manner (i.e. react prosocially). Pain scenes are pervasive, diverse and habitual sights in daily life^7^ and prosocial behavior has a cost^1, 7, 17, 18^. Whether to help, ignore, escape or even experience schadenfreude when seeing another in pain may depend on the estimation of the authenticity of the pain, the meaning to the observer, the relationship between the sufferer and the observer and the physical and social context^1, 7^. For example, a recent fMRI study revealed that subjects were more willing to help ingroup members than outgroup members^19^. Other research has shown that perceived fairness^20^, social stigma^21, 22^, and the familiarity and closeness between empathizer and empathizee^23, 24^ have an impact on empathy. Together, these studies suggest a screening process during empathy which checks the emotional surroundings and selects information which is important or meaningful for further processing. To date, surprisingly little attention has been given to the study of how and when this screening process occurs.

There is a considerable literature focusing on the neural mechanisms underpinning empathy, among which event-related potential (ERP) studies provide an outstanding perspective on exploring the temporal dynamic of empathy. Fan and Han used a convinced paradigm in which they required participants to perform either a pain judgment task or a hands counting task while watching pictures or cartoons depicting hands in pain^25^. They found that visual stimuli induced early and late ERP components that differentiated pain from neutral conditions. The early component, frontally distributed at 140 ms after stimulus presentation, was modulated by the reality of the stimuli, regardless of the task demand, hence corresponding to the affective sharing component^25^. In contrast, the late component, appearing over the central-parietal areas after 380 ms, was only evident when attending to pain cues (i.e., the pain judgment task), and hence was considered a cognitive evaluation component^25^. These findings have been confirmed by subsequent research^26–30^. Based on this research, it can be inferred that the “screening process” likely occurs during the late cognitive evaluation period. However, critical questions remain about this speculation. First, some indirect evidence has shown that one may estimate another’s pain in an automatic fashion. For instance, contextual reality^25^ and racial bias^27, 29, 31^ modulate the early automatic ERP component of pain empathy. The idea of an automatic screening mechanism is reasonable since it has great evolutionary benefit for the observer (e.g., alleviating cognitive load). In addition, ERP components, especially the late positive component, are commonly a mixture of many spatially or temporally overlapping neural activities (P3 and LPP subcomponents in the case of the late positivity) that cannot be separated from each other by traditional ERP methods^32, 33^. Thus, the specific time window during which the screening process occurs remains unclear. Direct evidence through the examination of brain activity during empathy is needed.

**Figure 1:**
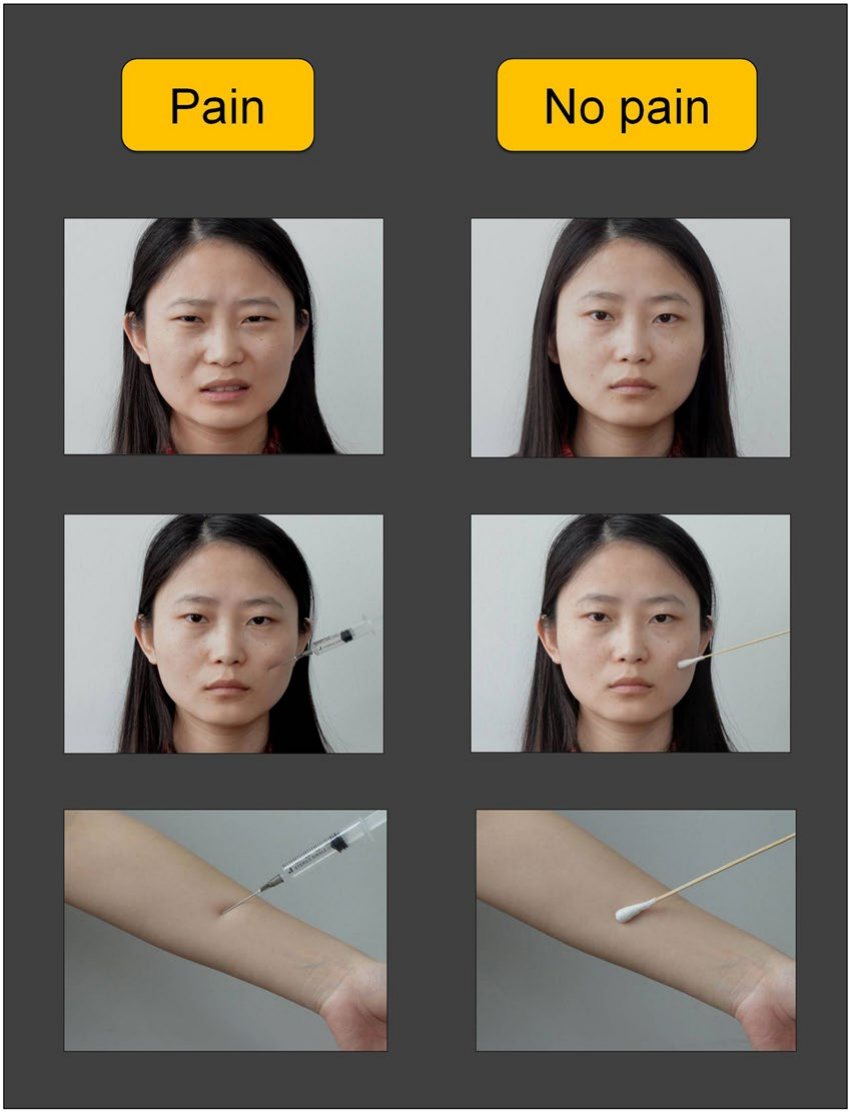
Experimental design and stimuli. A 2 (condition: pain vs. no-pain) × 3 (stimulus category: facial expressions, face pictures and arm pictures) within-subject factorial design was used. Examples of the visual stimuli are shown here. Top panel: painful expression vs. neutral expression, Middle panel: needle-penetrated face vs. Q-tip-touched face and Bottom panel: needle-penetrated arm vs. Q-tip-touched arm.

The present study used ERP to directly measure brain activity during the experience of empathy. Three different categories of painful pictures were used as empathy-eliciting stimuli: a painful facial expression, a needle-penetrated face and a needle-penetrated arm (Fig. 1). A neutral expression, a face touched with a Q-tip and an arm touched with a Q-tip were used as respective controls (i.e., no-pain scenes). All stimuli has previously been used to induce vicarious pain^10, 21, 26, 34, 35^. The painful expression and the needle-penetrated arm are more natural stimuli that may be experienced in daily life or in clinical settings, while a needle-penetrated face with a neutral expression is a more artificial stimulus, specifically designed for the laboratory, and likely not encountered in actual life settings. The needle-penetrated face accompanied by a neutral expression is perceived as a no-pain stimulus, and ERPs would not differentiate between the needle-penetrated face and a Q-tip-touched face. The aim of the current study was to explore the empathy screening process through ERP components. We expected that the ERP components would distinguish a painful expression from a neutral expression, a needle-penetrated arm from a Q-tip-touched arm, but would not distinguish between a needle-penetrated face and a Q-tip-touched face with a neutral expression. In addition, a temporospatial two-step principle component analysis (PCA) approach was applied to isolate the ERP components of such a process. Temporospatial PCA is a promising tool to capture variance across electrode sites and time points to separate latent components that may be conflated in traditional trial-averaged waveform measures of ERPs^32, 33, 36^.

## Results

### ERPs

**Figure 2:**
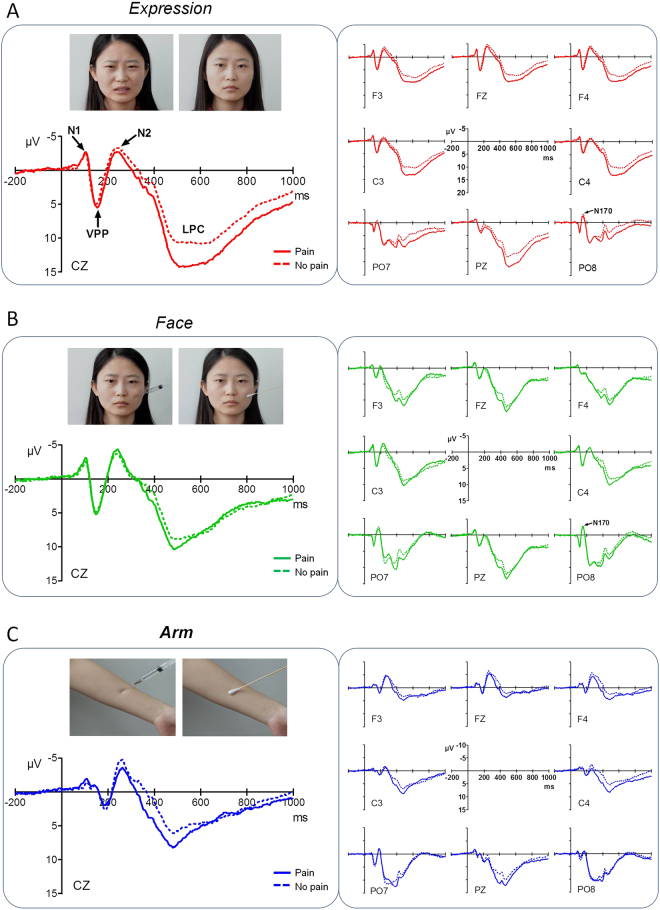
Grand average ERPs in response to three stimulus categories under pain and no-pain conditions at frontocentral (F3, F4, Fz, C3, C4 and Cz) and parieto-occipital (Pz, PO7 and PO8) electrode sites. All stimuli elicited similar components. At frontocentral sites, an N1 component is apparent, followed by a VPP component, an N2 component, and a long lasting LPC while at temporo-occipital sites, a posterior P1 component and an N170 component are elicited. Negative amplitudes are plotted upwards. (**A**) ERPs in response to painful expressions (red solid lines) and neutral expressions (red broken lines). (**B**) ERPs in response to needle-penetrated faces (green solid lines) and Q-tip-touched faces (green broken lines). (**C**) ERPs in response to needle-penetrated arms (blue solid lines) and Q-tip-touched arms (blue broken lines).

**Figure 3:**
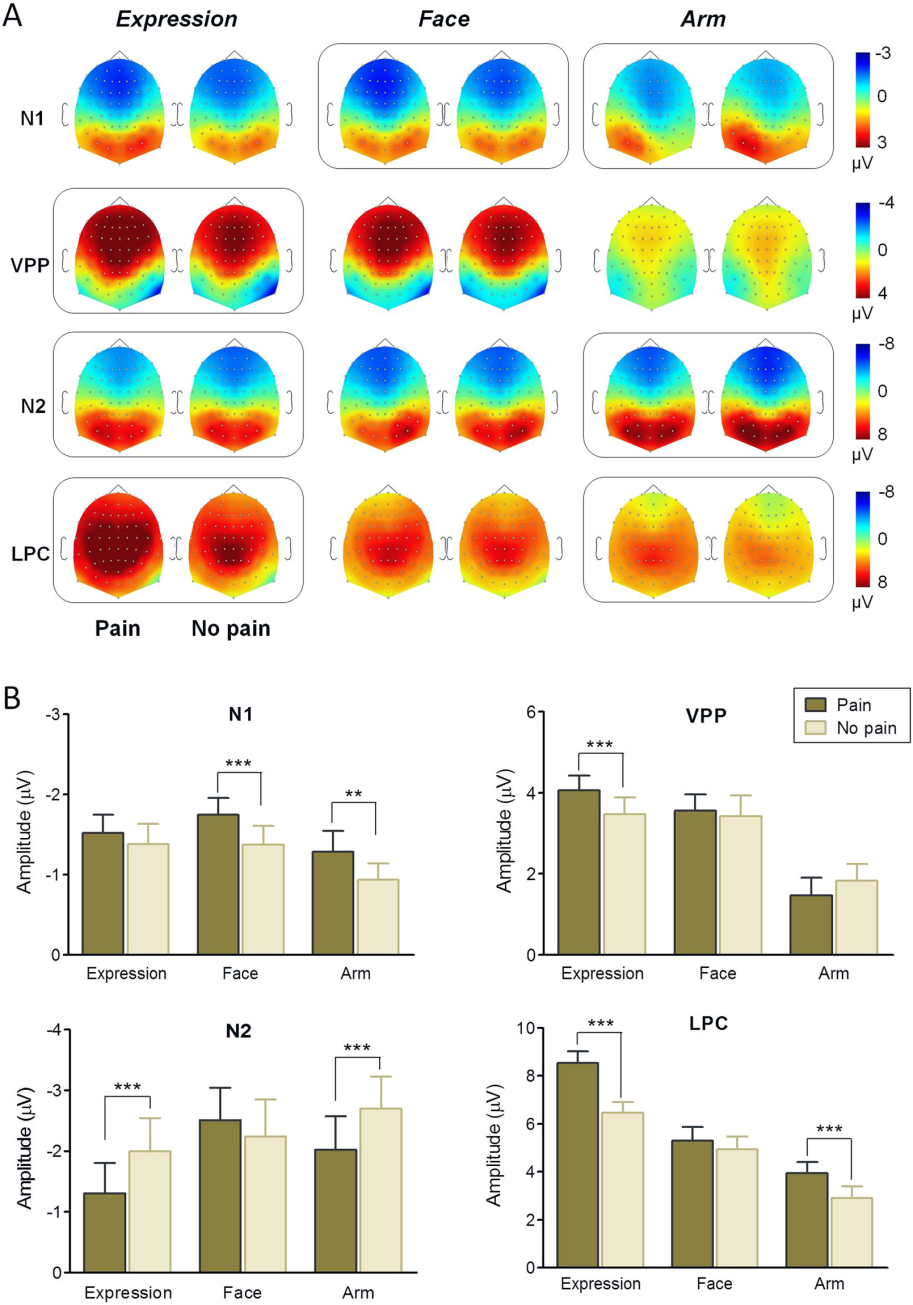
Topographical maps and statistical results of ERP components. (**A**) Scalp topographies of N1, VPP, N2, and LPC components (from top to bottom), plotted separately for each experimental condition (from left to right: painful expressions, neutral expressions, needle-penetrated faces, Q-tip-touched faces, needle-penetrated arms and Q-tip-touched arms). Boxed plots indicate a significant difference in ERP amplitude revealed by a Bonferroni-corrected *t* test. (**B**) Post hoc comparisons on condition × category interaction effects for N1, VPP, N2, and LPC amplitudes were conducted separately. Asterisks indicate significant differences in amplitude. **P < 0.01; ***P < 0.001. Error bars represent standard errors of the mean.

The grand average ERPs at electrodes of interest (F3, F4, Fz, C3, C4, Cz, Pz, PO7 and PO8) and scalp topographies prior to PCA are illustrated in Figs 2 and 3A. Visual stimuli of the three categories (facial expressions, face pictures and arm pictures) elicited similar ERP components. At frontocentral sites, an N1 component was apparent, followed by a vertex positive potential (VPP), an N2 component, and finally a long lasting late positive complex (LPC). At temporo-occipital sites, posterior P1 and N170 components (see Supplementary Information) were seen.

#### N1

Painful scenes elicited larger N1 potentials than neutral scenes, and N1 amplitudes were higher in response to face-containing pictures (i.e., facial expressions and face pictures) than to arm pictures (expressions vs. arms, *P* < 0.001; faces vs. arms, *P* < 0.001). A 3-way ANOVA on N1 amplitude revealed a significant main effect of condition [*F*(1, 276) = 56.18, *P* < 0.001, *η*_*P*_^2^ = 0.17], stimulus category [*F*(2, 552) = 25.70, *P* < 0.001, *η*_*P*_^2^ = 0.09], and laterality [*F*(2, 276) = 4.34, *P* = 0.014, *η*_*P*_^2^ = 0.03], and a significant interaction between category × laterality [*F*(4, 552) = 5.13, *P* < 0.001, *η*_*P*_^2^ = 0.04]. Post hoc analysis revealed significant differences between the needle-penetrated face and the Q-tip-touched face (*P* < 0.001), and between the needle-penetrated arm and the Q-tip-touched arm (*P* = 0.001; Fig. 3A and B). No differences were observed between painful expressions and neutral expressions (*P* > 0.999; Fig. 3A and B). Interestingly, when gender was taken into consideration, the early distinction between the needle-penetrated face and the Q-tip-touched face, and between the needle-penetrated arm and the Q-tip-touched arm were only found in females, but not in males, as revealed by the significant gender × condition × category interaction effect [*F*(1.97, 545.86) = 8.82, *P* = 0.005, *η*_*P*_^2^ = 0.02].

The 3-way ANOVA on N1 latency revealed a significant main effect of stimulus category [*F*(1.64, 447.30) = 5.29, *P* = 0.009, *η*_*P*_^2^ = 0.02]. Post hoc comparisons indicated that face-containing pictures were detected faster than arm pictures (expression vs. arm, *P* = 0.041; face vs. arm, *P* = 0.006). In addition, the condition × category interaction [*F*(1.99, 547.98) = 4.77, *P* = 0.009, *η*_*P*_^2^ = 0.02] was significant.

#### VPP

The VPP showed a greater amplitude in the pain condition relative to the no-pain condition. Face-containing pictures produced marked enhancement in the amplitude of the VPP over arm pictures (expression vs. arm, *P* < 0.001; face vs. arm, *P* < 0.001). The 3-way ANOVA for the VPP amplitude yielded a significant main effect of condition [*F*(1, 276) = 22.37, *P* < 0.001, *η*_*P*_^2^ = 0.07] and category [*F*(1.58, 436.79) = 130.93, *P* < 0.001, *η*_*P*_^2^ = 0.32], and a significant interaction effect of condition × category [*F*(1.79, 493.27) = 8.62, *P* < 0.001, *η*_*P*_^2^ = 0.03]. Post hoc comparisons revealed a significant difference between painful expressions and neutral expressions (*P* < 0.001; Fig. 3A and B). No differences were found for the other two (face pictures and arm pictures) stimulus categories (needle-penetrated face vs. Q-tip-touched face, *P* > 0.999; needle-penetrated arm vs. Q-tip-touched arm, *P* > 0.999; Fig. 3A and B).

For the VPP latency, the 3-way repeated measures ANOVA revealed a significant main effect of category [*F*(1.28, 291.63) = 291.63, *P* < 0.001, *η*_*P*_^2^ = 0.51], and a significant condition × category interaction effect [*F*(1.92, 529.35) = 4.77, *P* < 0.001, *η*_*P*_^2^ = 0.04). Similar to the results for the N1 component, face-containing pictures elicited shorter VPP latencies than arm pictures (expression vs. arm, *P* < 0.001; face vs. arm, *P* < 0.001; Fig. 2). In addition, there was a significant difference between painful expressions and neutral expressions (painful expression > neutral expressions, *P* < 0.001), whereas no differences were found for the other two (face pictures and arm pictures) stimulus categories (needle-penetrated face vs. Q-tip-touched face, *P* > 0.999; needle-penetrated arm vs. Q-tip-touched arm, *P* > 0.999).

#### N2

The N2 component showed a positive shift in the pain condition compared to the no-pain condition. Facial expression pictures elicited a more positive N2 than face pictures and arm pictures (expression vs. face, *P* < 0.001; expression vs. arm, *P* < 0.001; Fig. 3). In a 3-way repeated measures ANOVA of N2 amplitude, there were significant main effects of condition [*F*(1, 276) = 39.93, *P* < 0.001, *η*_*P*_^2^ = 0.13], category [*F*(1.58, 436.65) = 19.66, *P* < 0.001, *η*_*P*_^2^ = 0.07], and laterality [*F*(2, 276) = 4.76, *P* = 0.009, *η*_*P*_^2^ = 0.03], and significant interaction effects of condition × category [*F*(1.98, 545.41) = 28.00, *P* < 0.001, *η*_*P*_^2^ = 0.09], condition × laterality [*F*(2, 276) = 3.43, *P* = 0.034, *η*_*P*_^2^ = 0.02], category × laterality [*F*(3.16, 436.65) = 6.07, *P* < 0.001, *η*_*P*_^2^ = 0.04], and condition × category × laterality [*F*(3.95, 545.41) = 3.10, *P* = 0.016, *η*_*P*_^2^ = 0.02]. Post hoc comparisons indicated that N2 amplitudes were significantly different between painful expressions and neutral expressions (*P* < 0.001, Fig. 3B), and between needle-penetrated arms and Q-tip-touched arms (*P* < 0.001, Fig. 3B). No difference in N2 was observed between needle-penetrated faces and Q-tip-touched faces (*P* = 0.128, Fig. 3B). The pain-related N2 amplitude modulation was significant at midline and right hemisphere electrodes (both *P*s < 0.001), but was not significant at left hemisphere electrodes (*P* = 0.463).

There was a significant main effect of category on N2 latency [*F*(1.80, 495.49) = 71.34, *P* < 0.001, *η*_*P*_^2^ = 0.21]. The N2 latencies elicited by facial expression pictures were longer than those elicited by face pictures but shorter than those elicited by arm pictures (all *P*s < 0.001).

#### LPC

The 3-way ANOVA of LPC revealed significant main effects of condition [*F*(1, 276) = 221.43, *P* < 0.001, *η*_*P*_^2^ = 0.45] and category [*F*(1.94, 535.98) = 497.80, *P* < 0.001, *η*_*P*_^2^ = 0.64], and significant interaction effects of condition × category [*F*(1.87, 516.39) = 55.74, *P* < 0.001, *η*_*P*_^2^ = 0.17), condition × laterality [*F*(2, 276) = 5.15, *P* = 0.006, *η*_*P*_^2^ = 0.04] and category × laterality [*F*(3.88, 535.98) = 3.83, *P* = 0.005, *η*_*P*_^2^ = 0.03]. Post hoc analyses showed that the pain vs. no-pain difference in LPC existed in expression pictures and arm pictures (both *P*s < 0.001), but not in face pictures (*P* = 0.058).

### PCA results

**Figure 4:**
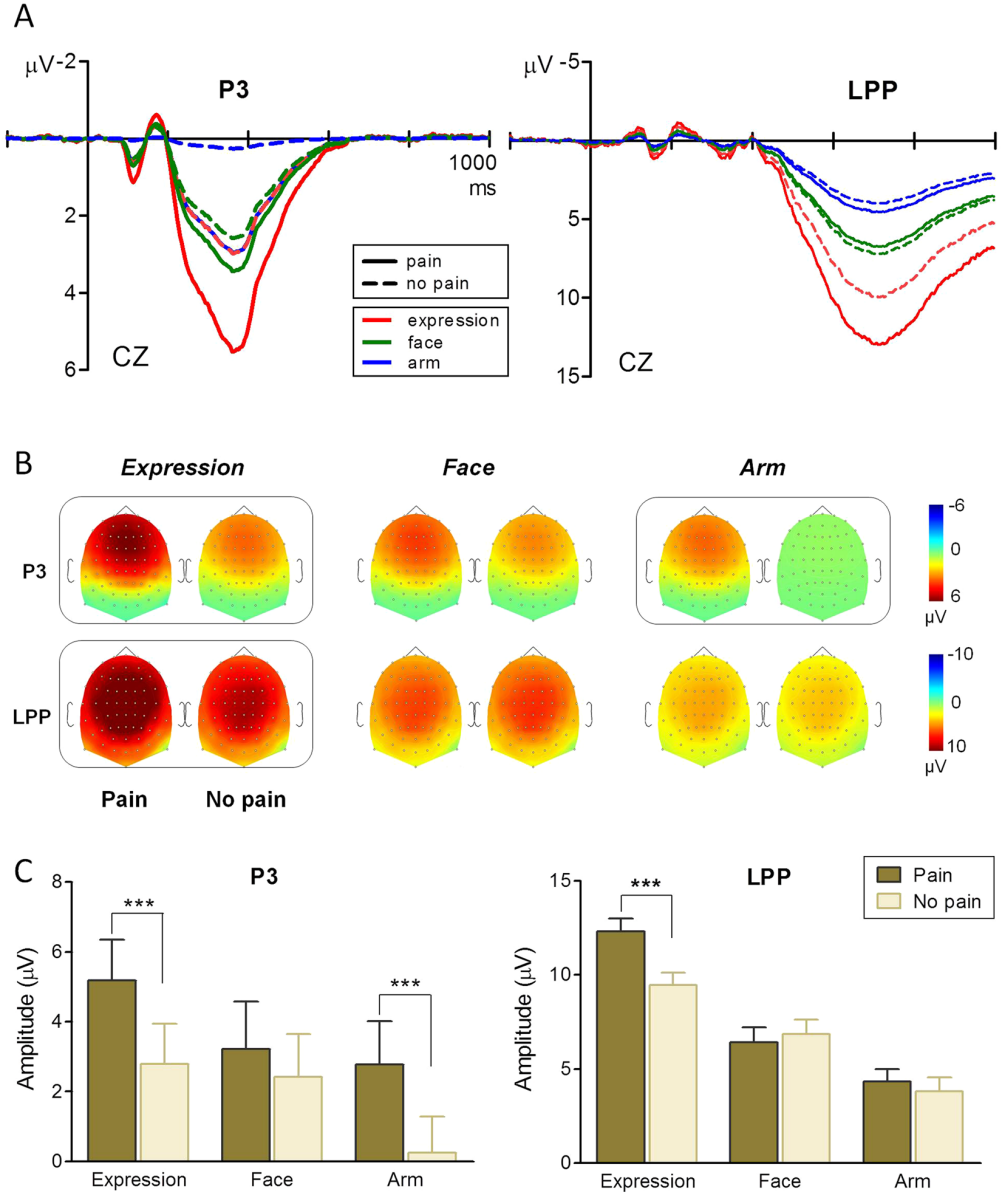
PCA derived P3 and LPP components. (**A**) Virtual PCA derived P3 (left) and LPP (right) component waveforms for each experimental condition. (**B**) Scalp topographies of P3 (upper panel) and LPP (lower panel) components, plotted separately for each experimental condition (from left to right: painful expressions, neutral expressions, needle-penetrated faces, Q-tip-touched faces, needle-penetrated arms and Q-tip-touched arms). Boxed plots indicate a significant difference revealed by a Bonferroni-corrected *t* test. (**C**) Post hoc comparisons on condition × category interaction effects for P3 (left) and LPP (right) components. Asterisks indicate significant differences in amplitude. ***P < 0.001. Error bars represent standard errors of the mean.

The LPC component was divided into two separate subcomponents by temporospatial PCA: P3 and LPP. The microvolt rescaled factor scores of the PCA-derived P3 and LPP components are presented in Fig. 4A. Two-way ANOVAs were conducted on these components, to better illustrate the empathic process over the long lasting late component (see Fig. 4B and C).

The P3 amplitudes were greater in response to painful scenes than to neutral scenes, and face-containing pictures elicited greater P3 amplitudes than arm pictures (expression vs. arm, *P* < 0.001; face vs. arm, *P* = 0.035; Fig. 4B and C). The 2-way ANOVA revealed a significant main effect of condition [*F*(1, 30) = 42.37, *P* < 0.001, *η*_*P*_^2^ = 0.59] and category [*F*(2, 60) = 12.03, *P* < 0.001, *η*_*P*_^2^ = 0.29], and a significant interaction effect between these variables [*F*(2, 60) = 6.26, *P* = 0.003, *η*_*P*_^2^ = 0.17]. Post hoc analysis revealed a significant pain-related difference between painful expressions and neutral expressions, and between needle-penetrated arms and Q-tip-touched arms (both *P*s < 0.001; Fig. 4B and C). No significant difference was found between the needle-penetrated face and the Q-tip-touched face (*P* = 0.622; Fig. 4B and C).

Painful scenes elicited considerably greater LPP components than neutral scenes. Expression pictures elicited the largest LPP, while arm pictures elicited the smallest LPP (all *P*s < 0.001). In the 2-way ANOVA for the LPP amplitude, there were significant main effects of condition [*F*(1, 30) = 15.85, *P* < 0.001, *η*_*P*_^2^ = 0.35] and category [*F*(2, 60) = 61.02, *P* < 0.001, *η*_*P*_^2^ = 0.67], and a significant condition × category interaction effect [*F*(2, 60) = 10.90, *P* < 0.001, *η*_*P*_^2^ = 0.27]. Post hoc comparisons revealed that the pain effect only existed in the expression category (*P* < 0.001; Fig. 4B and C), suggesting long lasting empathic processing for pain expression.

### Behavioral performance

Table 1 summarizes the mean RT and accuracy rate under each category and condition. Participants responded faster to pain pictures than neutral pictures [2-way ANOVA, condition effect: *F*(1, 30) = 19.52, *P* < 0.001, *η*_*P*_^2^ = 0.39] and took more time to judge expression pictures than to judge face and arm pictures [all *P*s < 0.001; category effect: *F*(2, 60) = 30.12, *P* < 0.001, *η*_*P*_^2^ = 0.50].

**Table 1:** Participants’ mean reaction time and accuracy rate of pain judgment task in each experimental condition.

Stimulus category	Condition	
	Pain	No pain
Reaction time (ms)		
Expression	756.69 (191.72)	784.40 (196.91)
Face	642.82 (152.78)^***#^	721.93 (249.48)
Arm	635.55 (165.02)^***^	686.96 (169.79)
Accuracy rate (%)		
Expression	91.85 (6.31)^#^	94.80 (2.76)
Face	97.53 (2.90)^***^	97.27 (1.92)
Arm	98.39 (1.27)^***^	98.11 (1.70)

Values are mean (standard deviation). ***P < 0.001, compared with painful expression. ^#^ *P* < 0.05, compared with corresponding no pain condition.

Participants were less accurate in judging painful scenes than neutral scenes, and also less accurate in judging expressions than faces and arms (all *P*s < 0.001). The 2-way repeated measures ANOVA revealed significant main effects of condition [*F*(1, 30) = 4.89, *P* = 0.035, *η*_*P*_^2^ = 0.14] and category [*F*(1.37, 41.18) = 40.80, *P* < 0.001, *η*_*P*_^2^ = 0.58], and a significant interaction effect of condition × category [*F*(1.35, 40.61) = 5.71, *P* = 0.014, *η*_*P*_^2^ = 0.16].

### Correlation analyses

Correlation analyses showed no associations between electrophysiological measures and any of the psychometric measures or behavioral performance.

## Discussion

The present study examined brain activity induced by empathy-eliciting painful scenes through the combination of ERP and PCA techniques. There were two main findings. First, a novel brain screening process during empathy processing for pain (i.e., N2 and P3 components) was discovered and is responsible for distinguishing between true and false pain scenarios. Second, face stimuli had priority over arm stimuli in early pain empathy processing, represented by shorter latencies and higher amplitudes of N1 and VPP components.

The painful expression and the needle-penetrated arm stimuli used in the current study are natural scenarios witnessed in daily life, and as such they can be considered to reflect true pain. In contrast, a neutral expression in response to a prick in the face with a needle is unnatural and thus the pictures depicting a needle-penetrated face are considered to reflect false pain. In the present study, a 2 (pain vs. no-pain) × 3 (facial expression vs. face pictures vs. arm pictures) within-subject design was adopted, revealing different temporal dynamics of neural activity between the observation of pain and no-pain pictures as well as between true and false pain pictures. Pain vs. no-pain information is differentiated in the very early ERP components, i.e., the N1/P1 for the face and arm pictures categories and the VPP/N170 for the facial expression category while the mid-latency ERP components, N2 and P3, played key roles in differentiating true from false situations. That is, N2 and P3 amplitudes were more positive in response to painful scenarios only in the facial expression (grimace) and arm picture (needle-penetrated arm) categories but not in the face category. Thus, the N2 and P3 activity constituted a crucial screening operation during empathy processing for pain. Finally, the long-latency component (the LPP) may be more associated with socioemotionally salient stimuli, as a larger LPP amplitude was exclusively observed in the facial expression category. The temporal characteristics of the components (N1/P1, VPP/N170, N2, P3, and LPP) triggered by empathy-eliciting scenes in the current study are consistent with previous findings^25, 29, 30, 37^. More importantly, we extended these findings by indicating that a judging process or a screening mechanism exists during empathy for pain, comprising of N2 and P3, and responsible for differentiating true from false pain situations.

The current study demonstrated that environmental pain information can be processed as early as 100 ms following stimulus onset (i.e., the N1/P1 component). The N1 and P1 components are widely accepted as signatures of early sensory processing and prior ERP studies have shown them to be sensitive to emotional content^32, 38^. The pain-related modulation of N1/P1 components may be interpreted as early differentiation between pain and no-pain processing. However, prior research has reported conflicting results on the pain effect on early ERP components. Some authors have reported a significant pain effect on N1/P1 amplitude^29, 30, 37^, while others have found no effect^25, 27, 31^. The controversial results may be due to different empathy-eliciting stimuli (e.g., painful expressions, injured body parts, etc.) used in these studies as well as different ERP component isolation methods. In general, observing injured body parts produces a significant N1/P1 modulation, as in the present study. It may be that injured body parts are more self-relevant than painful facial expressions. That is, when viewing an injured limb, foot, or other body part, observers may experience vicarious pain from the first-person perspective, thus generating a rapid aversive response.

An interesting finding in our study was that only females can differentiate pain vs. non-pain pictures in the N1 time window, suggesting that women were more sensitive in detecting pain-related information than men. This was consistent with a recent EEG research in which females show stronger mu suppressions (a robust biomarker of sensorimotor resonance in empathy) than males when watching painful pictures^39^, which indicated that females were more easily to imagine themselves into the painful situations. Gender difference is an interesting topic with regard to empathy. Although it is well accepted that females are more empathic than males in general, previous studies using different methods revealed inconsistent findings^20, 40–42^. Evidence has shown that females score higher than males in both trait empathy and state empathy^40, 43–45^. However, an influential meta-analysis has revealed no gender-specific activation in previous empathy fMRI studies^42^. In the present study, we found that females had faster brain processing speed in response to empathy-eliciting situations, providing evidence that females may be more empathic than males.

In line with previous face perception studies, the current results showed that face processing has priority over body-part processing. The early ERP components (N1/P1, VPP/N170) to face stimuli were faster and larger than those to arm stimuli. Face-driven modulation of N1/P1 amplitudes is controversial and such a modulation is commonly considered not specific to face perception^46, 47^. However, the enhanced amplitude and shortened latency in response to face-containing pictures reflects face-related facilitation of early visual processing. The N170 component and its positive vertex counterpart (VPP component) are well-accepted electrophysiological markers for face perception, as they both demonstrate larger amplitudes in response to faces than non-face stimuli^46^. Faces convey a wealth of information (including but not limited to sex, age, attractiveness, emotional state, and focus of attention) that is necessary for successful social interaction^48^. As social beings, humans are sensitive to social information^49^. The current findings indicate that cognitive resources are allocated preferentially to faces demonstrating pain. This finding, while preliminary, suggests that face-containing stimuli may produce stronger empathic effects than non face-containing stimuli^50^.

The current study revealed a screening process (comprised of N2 and P3) that discriminates true from false pain situations, in agreement with our hypothesis. Previous studies have shown that N2 amplitude is sensitive to emotional stimuli and reflects selective attention towards biologically important events, while P3 is closely associated with attention resource allocation based on motivational salience^32, 38, 51^. N2-P3 waveform has also been described as a uniform complex that reflects orienting response^52, 53^. The modulation of N2 and P3 amplitudes in viewing other’s pain suggests that painful scenes capture more attentional resources than no-pain scenes. This attentional bias occurred only in real pain situations (i.e., painful expressions and needle-penetrated arms) but not in artificial painful scenes (i.e., needle-penetrated faces). These results support our hypothesis that observers tend to first perceive environmental cues that are painful or unpleasant, and then select true or meaningful situations for further processing. Consistent with this idea, previous studies have shown that in-group characteristics, especially race^19, 26, 31, 54–56^, relationship between the empathy dyad^24, 30^, and contextual characteristics such as stimulus reality^25, 34^ influence empathic neural activities. Mainly using fMRI method thereby leaving the screening process undiscovered, nevertheless, these studies suggest that observers select some parts of perceived pain cues into empathy processing based on interpersonal or environmental characteristics. Sessa *et al*. investigated the effect of race on pain empathy processing by asking white participants to observe pain or no-pain pictures of people from the same or different races^27^. The pain vs. no-pain difference was magnified in the same-race condition compared to the other-race condition in a time window (280–340 ms) analogous to the N2-P3 component (approximately 250–360 ms) in our study. These findings suggest a similar screening mechanism that discriminates own race from other race during empathy processing for pain.

Although N2 and P3 components are both associated with the process of selective attention, it has been proposed that they reflect different aspects of attention processing. The mid-latency N2 component (peak﻿ between 200~300 ms) is a semiautomatic or pre-attentive process^57^, whereas the P3 component (peak﻿ after 300 ms) is generally associated with controlled attention or conscious processing^32, 38^. In parallel with this idea, the N2 and P3 components are viewed as automatic and controlled processes, respectively in pain empathy studies. For example, Fan and Han’s initial study^25^ found that pre-P3, short-latency electrophysiological activity was not influenced by task demand. However, the pain effect on long-latency waveforms depended on whether attention was allocated to pain cues or not. Since both the N2 and P3 components are associated with the screening process, it is suggested that the screening process involves engagement of attention and occurs during a shift from automatic to conscious attention.

Attention plays a vital role in empathy^58^ and prosocial behavior^2^. The screening mechanism, working as a social information filter and an attention allocator, allows meaningful environmental cues (e.g., pain experienced by a family member) to enter into further empathic processing, while preventing meaningless cues (i.e., irrelevant or unreasonable information) from engaging resources. Due to limited cognitive and physical resources (e.g., attention, time and money), one must analyze and evaluate environmental cues before making a decision to help (approach) or to reject (avoid) a situation. Through the screening mechanism, observers generate empathic feelings as well as helping behavior towards the victims whose situations are judged as painful, distressing, or serious.

By means of a temporospatial PCA approach, the long LPC component was divided into two subcomponents: the P3 and the LPP. As discussed before, the P3 component is part of the screening process and reflected a controlled orienting response. The LPP waveform, however, exhibited a different modulation pattern. The pain effect on LPP was only observed in the expression category but not in the face or arm categories. The LPP indexes ongoing, elaborative processing (cognitive appraisal) of motivationally salient information^32, 38^. Facial expression is one of the most effective methods by which to communicate pain information, and is considered a credible method in conveying suffering^59^. As social beings, human have evolved a propensity to detect and interpret facial expressions of pain^60^. Consistent with this idea, Reicherts *et al*. found that pain expressions elicited larger LPP components than neutral expressions as well as other emotional facial expressions (i.e., happiness and fear)^61^. In the present study, the painful expression stimulus was more complicated and socioemotionally salient than the needle-penetrated arm stimulus indicating that emotional expressions capture sustained attention given their inherent motivational value.

The traditional measurement of average ERP is not capable of parsing components that are spatially or temporally overlapped, such as P3 and LPP. These two components are intrinsically different from each other as discussed above. However, in previous empathy ERP studies they are treated as one single component^25, 27–29, 31^. Temporospatial PCA is a powerful method that can decompose an ERP waveform into its underlying components^32, 33, 36^. In the present study, we for the first time applied this method to analysis empathic ERP data, and separate out P3 and LPP subcomponents from a mixed long-lasting late positive component (i.e., LPC). This may help us get a better understanding of temporal structure of the screening mechanism.

The present study has several limitations. First, we aimed to identify a neural process that differentiates true pain from false pain. However, instead of a true-false determination task a pain-no pain judgment task was used. Prior studies have shown that task demand can have a marked influence on pain empathic ERPs (mainly the late components)^25^. Under the framework of empathy study, empathic feelings were more easily induced with a pain-relevant task which draws attention to pain cues. A pain-irrelevant task, on the other hand, withdraws attention from these cues, thus weakening the pain empathy effect. Second, the arm pictures used in the current study have greater visual similarity than the face pictures. This discrepancy between stimuli may produce somewhat different levels of arousal between stimulus categories and modulate the face vs. arm electrophysiological activities, especially in early ERP components. However, the face effect is well accepted in socioemotional research^46^, and therefore, this discrepancy may not be a serious problem.

In conclusion, this work was the first to identify the existence and time course of a brain screening mechanism during human empathy. Our results demonstrate that more attention is allocated to pain cues when the cues are classified as true pain, accompanied by more positive N2 and P3 ERP components. Observers screen out false, meaningless information, only allowing true and meaningful information into further empathic processing. Our data also revealed a face priority in early empathic processing in brain activity, confirming that face-containing materials are efficient stimuli in eliciting social emotional experience.

## Methods

### Participants

The original sample consisted of 36 healthy Chinese college students recruited locally through advertisements (internet posts). Five students were excluded from further analysis due to either poor performance on the experimental task or excessive EEG artifacts. Thus the final sample consisted of 31 participants (13 males and 18 females, mean age 21.90 ± 1.90 years; See Supplementary Table 1 for demographic information and scale scores of the original and final samples). All subjects were right handed, and had normal or corrected-to-normal vision, and no current or prior history of chronic pain, neurological or psychiatric disorders or developmental disability (self-report). They gave written informed consent prior to participation and received ¥60 (about $10) as compensation. The study was approved by the Research Ethics Committee of the Institute of Psychology, Chinese Academy of Sciences, and was performed in accordance with relevant guidelines and regulations.

### Experimental design and visual stimuli

A 2 × 3 within-subject factorial design was used with the pain condition (pain vs. no-pain) as the first factor, and the stimulus category (facial expression, face pictures, and arm pictures) as the second factor. Accordingly, there were six types of stimuli (painful expression vs. neutral expression, needle-penetrated face vs. Q-tip-touched face, and needle-penetrated arm vs. Q-tip-touched arm). As exemplified in Fig. 1, the painful expression stimulus depicted a person displaying a wrinkled, grimacing, and eye-narrowed face (pain expression according to Prkachin and Solomon^62, 63^), while a neutral expression depicted a person with an expressionless face. The needle-penetrated face and the Q-tip-touched face were both neutral faces with the left cheek being pricked by a syringe needle or touched by a Q-tip, respectively. The needle-penetrated arm and the Q-tip-touched arm were images depicting a forearm that was penetrated by a syringe and touched by a Q-tip, respectively. These stimulus types are the most commonly used materials in the field of empathy research, and are well accepted as valid representations of others in pain^10, 21, 26, 34, 35^.

The visual stimuli consisted of 180 digital static color pictures, with 30 pictures of each of the six stimulus types. All photographs used in the current study were taken by the first author using 30 different participants. The detailed information of the production and validation of the photographs are described in Supplementary Information. All visual stimuli were presented on a 14 inch color monitor with a black background at a viewing distance of 80 cm (subtending a visual angle of 16.7° wide × 12.5° high). The resolution of the screen was 1366 × 768 pixels.

### Procedure

The experimental task was administered on a personal computer running E-prime 2.0 (Psychology Tools Inc., Pittsburgh, PA, USA) to control stimulus presentation, timing and response recording. Prior to the EEG recording session, participants received detailed instructions and were familiarized with the task via six practice trials (one trial per stimulus type). Stimuli used during practice trials did not appear during the experiment. The experiment was comprised of ten blocks. Each block contained 90 trials, involving 15 trials of each experimental condition. Thus, each picture was presented five times, once every two blocks. Stimuli were fully randomized between participants and pseudorandomized within blocks (no more than two trials of the same stimulus type were presented consecutively). Participants were allowed a one minute break between every two successive blocks.

Each trial began with a red fixation cross (visual angle, 0.7° wide × 0.7° high) appearing at the center of the screen for a random, variable duration (800–1600 ms, with a 100-ms jitter). A picture stimulus was then displayed for 250 ms, followed by a blank screen interval (no time limit), during which subjects were instructed to indicate whether or not the picture represented a pain situation by pressing one of two buttons (‘F’ and ‘J’, counterbalanced across participants). Reaction times (RTs) and accuracy were measured. Once a response was made, the trial ended and the next trial began.

### Questionnaires

Dispositional empathy was assessed by a Chinese version of the Interpersonal Reactivity Index (C-IRI)^64^. The IRI is a 28-item self-administered survey consisting of four subscales (perspective taking [PT], fantasy [FS], empathic concern [EC], and personal distress [PD])^65^. The PT and FS subscales assess cognitive aspects of empathy: the former assesses the tendency to adopt another’s perspective and the latter reflects emotional identification with fictional characters in books, movies, and plays. The EC and PD subscales assess other- and self-oriented empathy-related emotional reactions, respectively. Specifically, the EC describes a person’s tendency to have feelings of sympathy and concern for others, and the PD measures the extent to which someone feels unease as a result of witnessing another’s emotional distress. The C-IRI was translated from the IRI using back translation, and factor analyses show that the two versions consist of the same factor components.

Catastrophic thinking about pain was measured by the Chinese version of the Pain Catastrophizing Scale (HK-PCS)^66^. Pain catastrophizing is defined as ‘an exaggerated negative mental set brought to bear during actual or anticipated painful experiences’, and is an important psychological characteristic that contributes to the perception and experience of pain^67^. The PCS is a 13-item self-report instrument that assesses how frequently participants experience ruminative, alarmist or helpless thoughts during pain. The PCS yields a total score and three subscale scores (rumination, magnification, and helplessness). The HK-PCS is a Chinese translation of the PCS and has proven reliable and valid.

### Electroencephalographic (EEG) data recording and preprocessing

EEG was continuously recorded via a BioSemi ActiveTwo system (BioSemi Inc., Amsterdam, The Netherlands) from 64 active electrodes based on the international 10–20 system throughout the pain judgment task. Signals were referenced online to the CMS-DRL (common mode sense-driven right leg) ground and digitized at 1024 Hz with 0.01 Hz high-pass and 100 Hz low-pass filtering. Impedances were maintained below 5 KΩ. The electrooculogram (EOG) was monitored through bipolar electrodes positioned approximately 1 cm from the outer canthus of each eye (horizontal EOG) and above and below the left eye (vertical EOG). Two additional sensors for off-line referencing were placed on the mastoid bones.

Raw EEG data were preprocessed using EEGLAB toolbox^68^ under Matlab (version 8.3., The MathWorks Inc., Natick, MA). All data were offline down-sampled to 256 Hz, re-referenced to averaged mastoids and filtered with a 0.1 Hz high-pass filter and a 50 Hz notch filter. The continuous EEG was then segmented for each trial, beginning 200 ms prior to stimulus onset and continuing for 1,000 ms after onset. After baseline correction, individual epochs contaminated by large nonstereotyped artifacts (e.g., gross muscle activity) were identified and eliminated by visual inspection. Remaining stereotyped artifacts such as saccades, blinks, cardiac artifacts and tonic muscle noises were corrected using Independent Component Analysis (ICA) methods. Independent components representing such stereotyped artifacts were removed in accordance with published guidelines^69^. For the artifact-clean trials, only those with a correct response and reaction time ≤2,000 ms were entered for analysis. Participants with less than 120 trials per experimental condition were excluded from analysis. The mean number of trials of each experimental condition for all participants is listed in the Supplementary Information.

### Event-related Potential (ERP) components

Conventional ERP analyses were performed first to enable comparisons with previous empathy ERP studies. Average ERPs were computed time-locked to stimulus onset at each electrode for each type of stimulus (painful expression/neutral expression, needle-penetrated face/Q-tip-touched face, needle-penetrated arm/Q-tip-touched arm). In the resulting waveforms, component latency windows were determined based on visual inspection of the grand-average ERPs (Fig. 2) as well as prior research using the same paradigm. The following components were identified: temporo-occipital (O1-O2, PO7-PO8) P1 (90–120 ms) and N170 (130–210 ms); frontocentral (F3-Fz-F4, C3-Cz -C4) and parietal (P3-Pz-P4) N1 (90–120 ms, concomitant with the P1 component), vertex positive potential (VPP, 130–210 ms, concomitant with the N170 component), N2 (210–300), and a late positive complex component (LPC, 350–1000 ms). As an averaged mastoid reference was used in the present study, some of the posterior components (e.g., early posterior negativity) were not apparent^32^. The latency of each component was determined by the peak latency within a given time window. The amplitude of each component was derived from the average value of a 30 ms (for N1 and P1 components) or a 40 ms (for N170, VPP and N2 components) interval centered at the peak latency, with the exception of the LPC amplitude which was calculated as the mean voltage over 350–1,000 ms.

### Principal Component Analysis

To better tease apart the components contributing to the positive peak during the LPC time window, a two-step temporospatial PCA was conducted using the ERP PCA toolkit 2.50^70^ and following published guidelines^33, 36, 71^. A covariance matrix and Kaiser normalization were used for both the temporal and spatial PCA. Six ERP averages (i.e., painful expression, neutral expression, needle-penetrated face, Q-tip-touched face, needle-penetrated arm and Q-tip-touched arm) containing temporal information from the entire epoch (307 time points in total) as well as spatial information from all 64 electrode sites for each participant were entered into the data matrix for PCA. A temporal PCA with Promax rotation was performed first to analyze temporal variance and to maximize the initial separation of ERP components^36^. This first step used 307 time points as variables, and considered the combination of all 31 participants, 64 channels, and six conditions as observations. For data reduction purposes, seven temporal factors (TFs) were extracted based on Scree plots^72^ using the parallel test^73^. Thereafter, separate spatial PCAs with Infomax rotation were performed on each temporal factor retained in the previous step to reduce spatial dimensions of datasets. This step used the electrodes as variables with the combination of all participants, conditions and temporal factor scores as observations. Based on the result of the parallel test, three spatial factors (SFs) were extracted from each TF, yielding a total of 21 temporospatial factor combinations (TFSF).

Twelve factor combinations accounting for more than 1% of the variance each were retained for further analysis. These factors accounted for 80.30% of the variance in total. Two combinations were most consistent with the morphology of the P3 and LPP. Specifically, TF2SF1 (accounting for 22.13% of the variance) was maximal at electrode Fz with a peak latency at 363 ms, resembling the P3 component. TF1SF1 (accounting for 23.79% of the variance) was maximal at electrode CPz with a peak latency at 710 ms, resembling the LPP component. Thus, TF2SF1 and TF1SF1 were identified as the PCA-derived P3 and LPP, respectively, and were selected for statistical analysis. To directly assess timing and spatial voltage distributions, the factors were translated back into microvolt scaling.

### Statistical analysis

Statistical analyses were performed using STATISTICA (StatSoft Inc., Tulsa, OK). Electrophysiological data were visualized using GraphPad Prism 5.0 (GraphPad Software Inc., La Jolla, CA, USA) and Brainstorm (http://neuroimage.usc.edu/brainstorm). Behavioral data (RTs and accuracy rate) were subjected to a 2 (condition: pain vs. no-pain) × 3 (category: facial expressions, face pictures and arm pictures) repeated measures analyses of variance (ANOVA) in accordance with the experimental design. ERP latencies and mean amplitudes within the predefined time windows for specific locations were submitted to ANOVAs examining condition and category effects as well as topography dimensions (laterality: left, midline and right for N1, VPP, N2 and LPC; and left vs. right for P1 and N170). With respect to PCA factors, 2 (condition) × 3 (category) ANOVAs were conducted on TF2SF1 and TF1SF1 factor scores separately. To investigate gender effect on empathic neural processing, additional 2 (gender: male vs. female) × 2 (condition: pain vs. no-pain) × 3 (category: facial expressions, face pictures and arm pictures) repeated measures ANOVAs were conducted on 5 main ERP components (i.e., N1, VPP, N2, and PCA deprived P3 and LPP). For these additional ANOVAs, data were presented only when the 3-way interaction was significant. For all ANOVAs, the Greenhouse-Geisser correction was applied when appropriate. Bonferroni comparisons were carried out as post-hoc tests. Of primary interest were the main effects of, and the interactions between, condition and stimulus type. Of secondary interest, we examined the relationship between psychological measures (empathy trait, catastrophic thinking) or behavioral performance (accuracy, RTs) and electrophysiological activities (ERP latencies, amplitudes, and PCA factor scores) using Pearson correlations. After Bonferroni correction, a familywise alpha of 0.05 was applied.

### Data availability statement

The datasets generated during and/or analyzed during the current study are available from the corresponding author on reasonable request.

## Electronic supplementary material

Supplementary Information
