# Supplementary material for: A Screening Mechanism Differentiating True from False Pain during Empathy
Source: Sci Rep. 2017 Sep 13;7:11492. doi: 10.1038/s41598-017-11963-x (PMC5597602; doi:10.1038/s41598-017-11963-x)
Supplement: Supplementary file 1 — Supplementary Information [file 41598_2017_11963_MOESM1_ESM.pdf]

A Screening Mechanism Differentiating True from False Pain during Empathy

Ya-Bin Sun[1,2], Xiao-Xiao Lin[1,2], Wen Ye[1,2], Ning Wang[1,2], Jin-Yan Wang[1,2,*], Fei Luo[1,2]

[1] CAS Key Laboratory of Mental Health, Institute of Psychology, Chinese Academy of Sciences, Beijing, 100101, P.R. China

[2] Department of Psychology, University of Chinese Academy of Sciences, Beijing, 100049, P.R. China

* Corresponding wangjy@psych.ac.cn

Supplementary Methods

Visual stimuli

One hundred and eighty color photographs depicting hands or faces in pain or no pain conditions were employed as stimuli in the formal experiment. To create these visual stimuli, 33 Chinese college students (15 males and 18 females, mean age 23.10 ± 1.51 years) were recruited as actors or actresses from Capital Normal University. All of them provided written informed consent, agreed to transfer copyrights of photographs to the research group, and were monetarily compensated for their time. Prior to photographing, actors/actresses were informed about the aim of the study. Actors/actresses were asked to wear light-colored clothing, no facial makeup, and to push their hair back from the forehead (a hairband was allowed if necessary). During recording sessions, actors/actresses were given instructions describing the procedure and guiding them to pose or to make expressions according to the six experimental conditions. In the painful expression condition, actors/actresses were asked to imagine that they were receiving a needle injection and the experimenter gave a detailed description of the painful scene to support vivid imagination and/or presented photographs of prototypical pain expressions[1] if necessary. All actors/actresses displayed facial expressions of pain consistent with previously described core expressions of pain [2,3]. With respect to the remaining 5 conditions (i.e., neutral expressions, needle-penetrated faces, Q-tip-touched faces, needle-penetrated arms and Q-tip-touched arms), actors/actresses were asked to produce a neutral expression and remain still during recording. In the needle-penetrated face and Q-tip-touched face conditions, the neutral face was displayed with a syringe needle or a Q-tip touching

the left cheek, respectively. Actors/actresses were then asked to hold out their left arm with palms facing upwards. The experimenter pressed a syringe needle or a Q-tip touching to the inner side of the forearm, for the needle-penetrated arm and Q-tip-touched arm conditions respectively. For each of the above mentioned poses, actors/actresses were asked to remain still for 1s, during which photographs of each condition were taken. Only faces were shown in face-containing pictures (i.e., painful expressions, neutral expressions, needle-penetrated faces, and Q-tip-touched faces), and only forearms were shown in the arm-containing pictures (needle-penetrated arms and Q-tip touched arms). At least two photographs were taken for each condition. Pictures were taken from a third-person perspective using a digital color camcorder (LEGRIA HF R18; Canon Inc., Tokyo, Japan). Actors/actresses were seated approximately 1.5 m from the photographer against a pale grey background. After recording, all photographs were edited offline. Pictures were cropped to 1200 × 900 pixels. In order to maintain a high level of ecological validity, stimulus parameters (e.g. spatial frequency, complexity, luminance, contrast, etc.) were not equalized across conditions.

An additional 31 college students (16 males and 15 females, mean age 22.71 ± 2.36 years) were recruited to participate in rating the pictures. The raters were asked to observe the photographs and evaluate pain intensity on an 11-point numerical rating scale (0 = no pain at all, and 10 = the most imaginable pain), and to evaluate facial attractiveness on an 11-point scale (0 = not attractive at all, and 10 = the most attractive). Based on the pain intensity ratings, 180 digital static color pictures from

30 actors (15 males and 15 females, each person contributed one picture to each of the six conditions) were chosen as the final stimuli. The 30 actors had normal facial appearance, with a mean facial attractiveness rating of 5.09 ± 0.60. The mean and standard deviation of the pain intensity ratings were as follows: painful expression = 6.39 ± 0.59, neutral expression = 0.59 ± 0.19, needle-penetrated face = 5.98 ± 0.19, Q-tip-touched face = 0.65 ± 0.12, needle-penetrated arm = 5.64 ± 0.21, and Q-tip-touched arm = 0.50 ± 0.09, suggesting pain pictures were considered painful and no-pain pictures were considered neutral.

Supplementary Results

Trial number

The mean number of artifact-free trials was 132.29 ± 9.80 for painful expressions, 136.45 ± 5.81 for neutral expressions, 139.97 ± 6.95 for needle-penetrated faces, 140.23 ± 6.48 for Q-tip-touched faces, 141.55 ± 6.10 for needle-penetrated arms, and 141.39 ± 5.69 for Q-tip-touched arms. A 2 (condition: pain vs. no-pain) × 3 (category: expression, face pictures, arm pictures) repeated measures ANOVA revealed a significant main effect of condition [$F(1, 30)= 4.63$, $P = 0.040$, $\eta_P^2 = 0.13$] and category [$F(1.54, 33.37) = 33.37$, $P < 0.001$, $\eta_P^2 = 0.53$] and a significant interaction of condition × category [$F(1.56, 46.91) = 3.43$, $P = 0.040$, $\eta_P^2 = 0.10$).

P1 amplitude and latency

The 3-way ANOVA on P1 amplitude revealed a significant condition × category

interaction effect [F(1.99, 242.99) = 31.31, P < 0.001, η_P^2 = 0.20]. Post hoc analysis showed that P1 amplitudes were greater for face-containing pictures than arm-containing pictures under pain conditions (painful expression vs. needle-penetrated arm, P < 0.001; needle-penetrated face vs. needle-penetrated arm, P < 0.001), whereas an opposite pattern was revealed under no-pain conditions (neutral expression vs. Q-tip-touched arm, P < 0.001; Q-tip-touched face vs. Q-tip-touched arm, P < 0.001). Furthermore, a painful expression elicited a greater P1 amplitude than a neutral expression (P = 0.010). A significant category × laterality interaction effect [F(1.52, 185.81) = 18.33, P < 0.001, η_P^2 = 0.13] was also revealed, suggesting a P1 left hemisphere dominance for arm pictures (P = 0.002), whereas no hemisphere differences existed for the other two categories.

The 3-way ANOVA for P1 latency revealed a significant main effect of stimulus category [F(1.53, 187.73) = 44.07, P < 0.001, η_P^2 = 0.27] and a significant interaction effect of condition × category [F(1.98, 241.53) = 3.43, P = 0.034, η_P^2 = 0.034]. Post hoc comparisons found that the P1 latency was shorter in response to face-containing pictures than arm pictures (expression vs. arm, P < 0.001; face vs. arm, P < 0.001).

N170 amplitude and latency

A positive shift in the N170 component was present in response to pain scenes versus no-pain scenes. Face category pictures elicited significantly larger N170 components than arm pictures (P < 0.001). The 3-way ANOVA revealed a significant main effect of condition [F(1, 122) = 4.28, P = 0.041, η_P^2 = 0.03], a significant main

effect of category [$F(1.49, 181.33) = 13.84$, $P < 0.001$, $\eta_P^2 = 0.10$), a significant category × laterality effect [$F(1.49, 181.33) = 7.14$, $P = 0.003$, $\eta_P^2 = 0.06$], and a significant condition × category interaction effect [$F(2.00, 242.98) = 10.86$, $P < 0.001$, $\eta_P^2 = 0.08$). Post hoc comparisons found that the N170 elicited by a painful expression was greater than that elicited by a neutral expression ($P < 0.001$), while there was no pain-driven modulation in the other two categories (needle-penetrated face vs. Q-tip-touched face, $P = 0.863$; needle-penetrated arm vs. Q-tip-touched arm, $P = 0.266$).

The N170 latency was shorter in response to pain scenes compared to no-pain scenes. The expression stimuli-elicited N170 latency was the shortest, and the arm picture-elicited N170 latency was the longest (all *P*s < 0.001). In addition, the N170 latency was significantly shorter in the right hemisphere compared to the left hemisphere. The 3-way ANOVA on N170 latency revealed significant main effects of condition [$F(1, 122) = 6.68$, $P = 0.011$, $\eta_P^2 = 0.05$], category [$F(1.88, 229.93) = 93.30$, $P < 0.001$, $\eta_P^2 = 0.43$] and laterality [$F(1, 122) = 11.89$, $P < 0.001$, $\eta_P^2 = 0.09$], and significant interaction effects of condition × laterality [$F(1, 122) = 4.32$ $P = 0.040$, $\eta_P^2 = 0.03$] and category × laterality [$F(1.88, 229.93) = 7.49$, $P < 0.001$, $\eta_P^2 = 0.06$]. The condition × laterality post hoc analysis revealed that the pain effect on N170 latency was only significant in the right hemisphere ($P = 0.008$).

Supplementary References

1 Sun, Y. B., Wang, Y. Z., Wang, J. Y. & Luo, F. Emotional mimicry signals pain empathy as evidenced by facial electromyography. *Sci. Rep.* **5**, 16988-16988 (2015).

2 Prkachin, K. M. The consistency of facial expressions of pain: A comparison across modalities. *Pain* **51**, 297–306 (1992).

3 Prkachin, K. M. & Solomon, P. E. The structure, reliability and validity of pain expression: Evidence from patients with shoulder pain. *Pain* **139**, 267-274 (2008).

Supplementary Table 1. Demographic characteristics, trait empathy scores and pain catastrophic thoughts of participants in the formal experiment.

	Age (years)	Gender (male/ female)	Years of education	IRI				PCS		
				Perspective taking	Fantasy	Empathic concern	Personal distress	Rumination	Magnification	Helpless
All Subjects (36)	21.83 (1.90)	16/ 20	15.19 (1.31)	18.33 (3.66)	15.80 (4.17)	21.06 (3.68)	17.44 (3.43)	8.64 (2.77)	5.36 (2.46)	10.36 (4.76)
Remaining Subjects (31)	21.90 (1.90)	13/ 18	15.19 (1.22)	18.32 (3.48)	15.58 (4.32)	21.19 (3.46)	17.52 (3.57)	9.03 (2.71)	5.61 (2.45)	11.10 (4.48)

Values are mean (standard deviation). IRI = Interpersonal Reactivity Index. PCS = Pain Catastrophizing Scale.
